# Synthesis of aminoacylated *N*^6^,*N*^6^-dimethyladenosine solid support for efficient access to hydrolysis-resistant 3′-charged tRNA mimics

**DOI:** 10.1016/j.bmc.2014.09.054

**Published:** 2014-12-15

**Authors:** Sandro Neuner, Ronald Micura

**Affiliations:** Institute of Organic Chemistry and Center for Molecular Biosciences, CMBI, University of Innsbruck, Innrain 80-82, 6020 Innsbruck, Austria

**Keywords:** Nucleosides, Bioconjugates, Azide, Trimethyllysine, Oligonucleotide synthesis, Ribosomes

## Abstract

RNA-amino acid and RNA-peptide conjugates that mimic charged tRNA 3′-ends are valuable substrates for structural and functional investigations of ribosomal complexes. To obtain such conjugates, most synthetic approaches that are found in the literature make use of puromycin. This well available aminonucleoside antibiotic contains a dimethylamino group at the nucleobase and a methylated tyrosine that is connected via an amide linkage to the ribose moiety. To increase structural diversity, we present the synthesis of a *N*^6^,*N*^6^-dimethylated 3′-azido-3′-deoxyadenosine precursor that can be coupled to any amino acid. Further derivatization results in the solid support that is eligible for the preparation of stable 3′-aminoacyl- or 3′-peptidyl-tRNA termini with an amide instead of the natural ester linkage. The present work expands our previously established route that delivered a broad range of peptidyl-tRNA mimics to the corresponding counterparts with *N*^6^,*N*^6^-dimethylation pattern of the terminal adenosine (A76). This aspect is of significance to modulate the binding preferences of the mimics for ribosomal A- versus P-site.

## Graphical abstract

**Figure:**
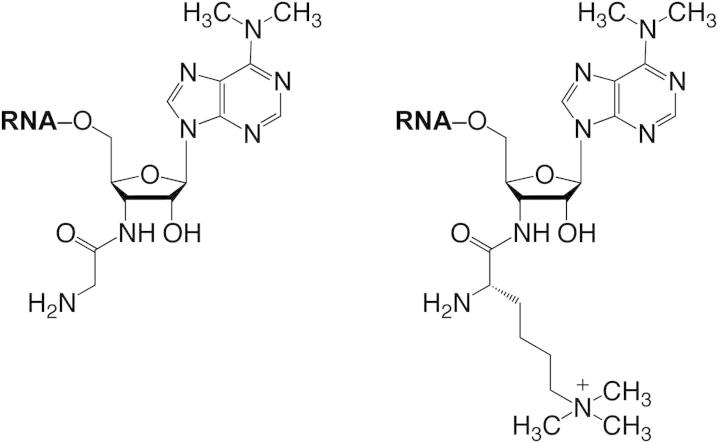

## 1 Introduction

High-resolution structures of the ribosome and its complexes with protein factors have become an indispensible basis to understand the intriguing functions of this cellular apparatus.^1^, ^2^, ^3^, ^4^, ^5^ They deliver fundamental insights through snapshots along the mechanistic road map of ribosome function and facilitate the design of genetic and biochemical experiments due to the knowledge of the three-dimensional positioning of the functional groups of interest.

**Figure 1:**
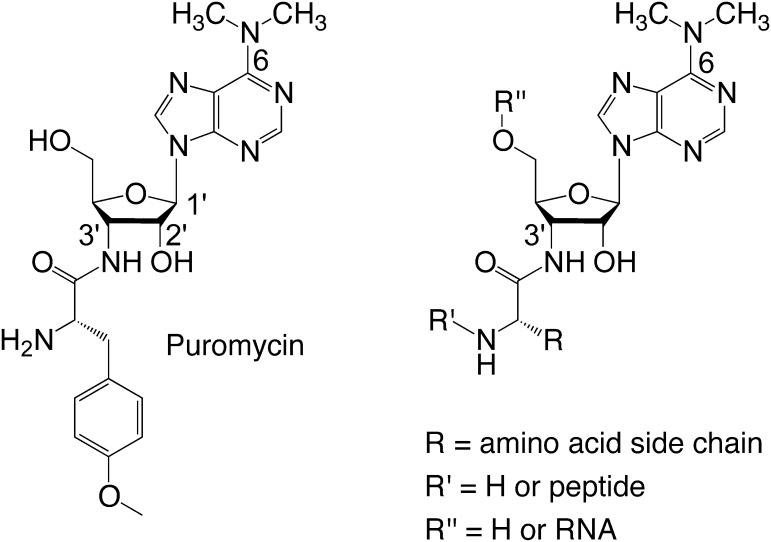
Structure of puromycin and related constitution of 3′-aminoacyl-tRNA mimics with hydrolysis-resistant 3′-ribose amide linkage, adenine dimethylation pattern, and any amino acid side chain.

For many X-ray crystallographic or cryo-electron-microscopic approaches, ribosome assembly with peptidyl- or aminoacyl-tRNA is needed. Such efforts can be severely hindered because of the easily hydrolyzable ester group that results in cleavage of the peptide moiety from the tRNA.^6^ These limitations, however, can be overcome by a stable linkage, for instance by 3′-amide-linked aminoacyl- or peptidyl-tRNA mimics that represent analogues to the naturally occurring antibiotic puromycin (Pmn) (Fig. 1).^6^, ^7^, ^8^ Such conjugates possess the propensity of higher stability during ribosome/tRNA assembly procedures, and at the same time, prevent ribose 2′-*O*/3′-*O* transesterification or transpeptidation. They are of immediate interest for the structural characterization of the diverse functional states passed through during the ribosomal elongation cycle and phenomena related thereto,^9^, ^10^, ^11^, ^12^, ^13^, ^14^, ^15^, ^16^, ^17^, ^18^, ^19^, ^20^ where mimics can be useful for A- and/or P-site substrate occupation. More recently, conjugates of that kind have been applied to elucidate peptide-mediated macrolide antibiotic resistance and ribosome stalling,^21^ and for investigations of selenocysteine biosynthesis.^22^

The synthesis of 3′-aminoacyl- and 3′-peptidyl-RNA conjugates is challenging and often represents a serious hurdle. Therefore, we and others have put significant efforts into novel synthetic strategies that rely on 3′-aminoacylamino-3′-deoxyadenosine modified solid supports and consecutive peptide and RNA solid-phase synthesis.^23^, ^24^, ^25^, ^26^, ^27^ More recent developments from our group have expanded the procedures to allow for site-specific methylation, phosphorylation and phosphonation of the RNA-peptide conjugates,^22^ and beyond that, use native chemical ligation to increase amino acid side chain flexibility.^28^, ^29^ All of our approaches so far pass a 3′-azido-3′-deoxyadenosine derivative as key intermediate, as originally shown in 2009.^26^ This intermediate allows to couple structurally diverse amino acids at a late stage of the synthesis, representing a significant advantage over most other synthetic strategies that make use of puromycin as key precursor,^11^, ^30^ thereby accepting the drawback that the amino acid at the junction is restricted to *O*-methylated tyrosine.

Another difference to puromycin derived tRNA mimics accounts for the dimethylation pattern at the exocyclic amino group of adenine. This methylation pattern has been discussed to influence A- versus P-site binding specificity of short CCA/CCPmn substrates.^31^ Unfortunately, efficient routes to obtain RNA conjugates with a terminal 3′-amino-3′-deoxy-*N*^6^,*N*^6^-dimethyladenosine and with flexibility concerning the nature of the connected amino acid are still lacking. The synthesis of this type of conjugates currently relies on the hydrolysis of puromycin which is cost-intensive and limited in terms of quantities.^32^ We therefore set out to create an alternative and convenient approach, exemplified here by the syntheses of the hydrolysis-resistant ACC(m^6^_2_A)-Gly and ACC(m^6^_2_A)-Lys(*N*^6^,*N*^6^,*N*^6^-(CH_3_)_3_) tRNA mimics.

## 2 Results and discussion

**Scheme 1:**
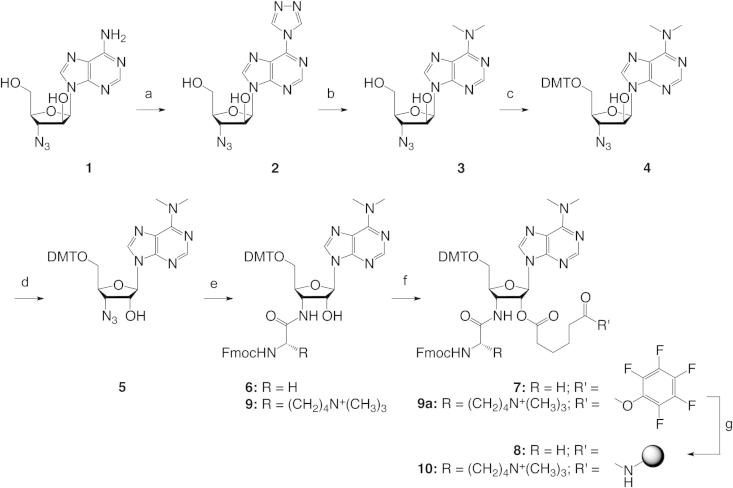
Synthesis of the modified solid supports **8** and **10**. Reaction conditions: (a) 3.5 equiv *N*,*N*′-bis[(dimethylamino)methylene]-hydrazine (BDMAMH) dihydrochloride, 2.5 equiv Et_3_SiCl in pyridine, reflux, 24 h; (b) 40% aqueous dimethylamine, in pyridine, rt, 1 h, (72%, over (a) and (b)); (c) 1.2 equiv DMT-Cl, in pyridine, 16 h, rt, 93%; (d) i. 1.6 equiv trifluoromethanesulfonyl chloride, 1.6 equiv DMAP, 2.8 equiv (iPr)_2_NEt in CH_2_Cl_2_, 30 min, 0 °C, 84%, ii. 5.5 equiv CF_3_COO^−^K^+^, 2.0 equiv 18-crown-6, 2.5 equiv (iPr)_2_NEt in toluene, 16 h, 80 °C, 81%; (e) i. H_2_/Pd, ethyl acetate, 22 h, rt, 92%, ii. 1.3 equiv Fmoc-Gly-OPfp in DMF, rt, 5 h, 95% or 1.5 equiv Fmoc-Lys(CH_3_)_3_-OBt in DMF, rt, 12 h, 27%; (f) 3 equiv or 2 equiv of adipic acid bis(pentafluorophenyl)ester for **6** and **9** respectively, 1 equiv DMAP in *N*,*N*-dimethylformamide/pyridine (1/1, v/v), rt, 1 h, 51% for **7**, (**9a** was used without isolation in the next step); (g) ∼1 equiv amino-functionalized polystyrene support (*GE Healthcare*, Custom Primer Support™ 200 Amino), pyridine, *N*,*N*-dimethylformamide, rt, 1 day, loading: 45 μmol/g for **8** and 18 μmol/g for **10**. DMAP = 4-(*N*,*N*-dimethylamino)pyridine, Fmoc = *N*-(9-fluorenyl)methoxycarbonyl, Pfp = pentafluorophenyl.

The starting point for our undertaking was 9-(3′-azido-3′-deoxy-β-D-arabinofuranosyl)adenine **1** (Scheme 1), which was synthesized in two steps from commercially available 9-(arabinofuranosyl)adenine based on a published procedure.^33^ Treatment of **1** with *N*,*N*′-bis[(dimethylamino)methylene]-hydrazine (BDMAMH) dihydrochloride^34^, ^35^ and TESCl in pyridine gave 9-(3-azido-3-deoxy-β-D-arabinofuranosyl)-6-(1,2,4-triazol-4-yl)purine **2**, which was converted quantitatively into 9-(3-azido-3-deoxy-β-D-arabinofuranosyl)-6-(dimethylamino)purine **3** with aqueous dimethylamine. Then, derivative **3** was transformed into the dimethoxytritylated compound **4**. Triflation of the arabinose 2′-OH resulted in intermediate **4a**, which was converted into the ribonucleoside **5** in diastereoselective manner by treatment with potassium trifluoroacetate and 18-crown-6-ether. Subsequently, Pd/C-catalyzed hydrogenation provided 9-(3-amino-3-deoxy-β-D-ribo-furanosyl)-6-(dimethylamino)purine **5a** as intermediate. Coupling with Fmoc-protected glycine furnished the amino acid linked building blocks **6**, which was further transformed into the pentafluorophenyl active ester **7** using adipic acid bis(pentafluorophenyl)ester^8^ as tether. Coupling of compound **7** to amino-modified polystyrene (*GE Healthcare*, Custom Primer Support™ 200 Amino) finally yielded the desired 3′-aminoacylamino-3′-deoxy-*N*^6^,*N*^6^-dimethyladenosine-functionalized solid support **8** (Scheme 1). To demonstrate the versatility of the approach we applied *N*^6^,*N*^6^,*N*^6^-trimethyl-L-lysine instead of glycine and obtained the corresponding solid support **10**.

In principle, solid supports **8** and **10** can be utilized for elongation of a peptide by automated solid-phase peptide synthesis based on *N*-(9-fluorenyl)methoxycarbonyl (Fmoc) protected amino acids, as we have shown previously on the analogous 3′-aminoacylamino-3′-deoxyadenosine-functionalized solid support series.^22^, ^26^, ^36^ Here, the support **8** was manually coupled with the corresponding Fmoc-protected amino acid using the tetramethyl-*O*-(1*H*-benzotriazol-1-yl)uronium (HBTU) reagent. Subsequently, solid supports **8** and **10** were directly supplied to solid phase RNA synthesis. Oligoribonucleotide assembly was based on 2′-*O*-[(triisopropylsilyl)oxy]methyl (2′-*O*-TOM)^37^, ^38^ protected nucleoside phosphoramidites and was performed on an oligonucleotide synthesizer following standard protocols. The introduction of the 5′-phosphate group at the 5′-end of RNA-peptide conjugates was accomplished as final cycle of the automated solid-phase synthesis by using a modified phosphoramidite building block prepared according to the literature.^39^

**Figure 2:**
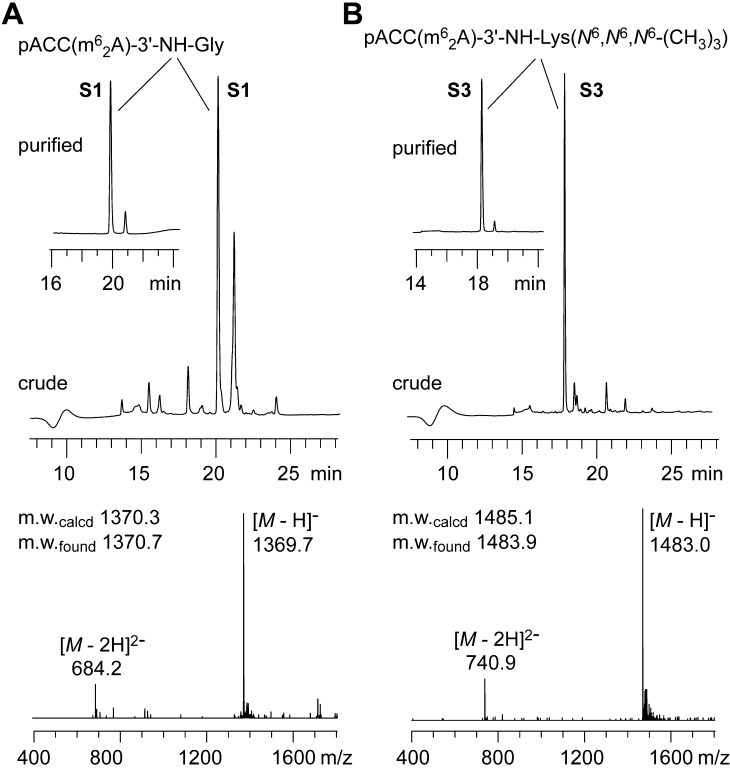
Synthesis of RNA-peptide conjugates based on the modified solid supports **8** and **10**. Anion-exchange HPLC profiles of crude and purified (insets) conjugates **S1** and **S3** (**A**,**B**; upper panels) and LC-ESI mass spectra of purified products (**A**,**B**; lower panels). Anion-exchange chromatography conditions: *Dionex* DNAPac®PA-100 (4 × 250 mm) column; temperature: 60 °C; flow rate: 1 mL/min; eluant A: 25 mM Tris·HCl (pH 8.0), 6 M urea; eluant B: 25 mM Tris·HCl (pH 8.0), 6 M urea, 500 mM NaClO_4_; gradient: 0–40% B in A within 25 min; UV detection at 260 nm.

The solid supports were treated with diethylamine in acetonitrile to eliminate the cyanoethyl and Fmoc protecting groups. The conjugates were then cleaved from the solid support using aqueous ammonia in ethanol under concomitant cleavage of the remaining acetyl protecting groups. Subsequent treatment with 1.0 M tetrabutylammonium fluoride trihydrate (TBAF·3H_2_O) in tetrahydrofuran at 37 °C overnight liberated the 2′-OH groups. This procedure resulted in high quality crude products which were analyzed by anion-exchange HPLC. The major peak of the HPLC profiles typically represented the desired conjugate which was purified on a semi-preparative column to achieve purities of more than 95%. Finally, the integrity of the products was analyzed by LC-ESI mass spectrometry and confirmed the expected molecular weights (Fig. 2). Following this approach, we prepared the short aminoacyl-RNA and dipeptide-RNA conjugates listed in Table 1.

**Table 1:** Selection of synthesized RNA-peptide conjugates

No	Sequence^a^	Amount (nmol)	m.w._calcd_ (amu)	m.w._found_ (amu)
**S1**	5′-p-ACC(m^6^_2_A-3′-NH)-Gly	420	1370.27	1370.69
**S2**	5′-p-GCACC(m^6^_2_A-3′-NH)-Gly	260	2021.31	2021.27
**S3**	5′-p-ACC(m^6^_2_A-3′-NH)-Lys(CH_3_)_3_	130	1485.13	1483.92
**S4**	5′-p-ACC(m^6^_2_A-3′NH)-Gly-Phe	600	1518.10	1517.92
**S5**	5′-pGCACC(m^6^_2_A-3′NH)-Gly-Phe	620	2168.49	2168.43

a For chemical structure of the nucleoside junction see Figure 1.

## 3 Conclusion

In this study, we have presented the efficient synthesis of the functionalized solid supports **8** and **10** for solid-phase synthesis of hydrolysis-resistant 3′-peptidyl-tRNA conjugates with puromycin-like *N*^6^,*N*^6^-dimethyladenosine pattern at the nucleoside junction. The novel aspects compared to our previous work and to work by others who use puromycin as tether, are given by the synthetic flexibility concerning the nature of the amino acid attached to the dimethylated adenosine and its convenient access from 9-(3′-azido-3′-deoxy-β-D-arabinofuranosyl)purine **1** in large quantities. Therefore, we significantly expand the scope of applications of these conjugates which are required to explore mechanistic details of ribosomal translation.^9^ In particular, these derivatives are promising to modulate the binding preferences of the mimics for ribosomal A- versus P-site in crystallographic studies.

## 4 Experimental

### 4.1 Solid support synthesis

#### 4.1.1 General remarks

Reagents were purchased in the highest available quality from commercial suppliers (Sigma–Aldrich, Acros, IRIS Biotech GmbH) and used without further purification. Organic solvents for reactions were dried overnight over freshly activated molecular sieves (4 Å). The reactions were carried out under argon atmosphere. ^1^H and ^13^C spectra were recorded on a Bruker DRX 300 MHz spectrometer. Chemical shifts (*δ*) are reported relative to tetramethylsilane (TMS) referenced to the residual proton signal of the deuterated solvent DMSO-*d*_6_ (2.50 ppm for ^1^H NMR spectra and 39.52 ppm for ^13^C spectra). The following abbreviations were used to denote multiplicities: s = singlet, d = doublet, t = triplet, m = multiplet, b = broad. Signal assignments are based on ^1^H-^1^H-COSY and ^1^H-^13^C-HSQC experiments. MS experiments were performed on a Finnigan LCQ Advantage MAX ion trap instrumentation (Thermo Fisher Scientific) with an electrospray ion source. Samples were analyzed in the positive- or negative-ion mode. Reaction control was performed via analytical thin-layer chromatography (TLC, Macherey-Nagel) with fluorescent indicator. Spots were further visualized using cerium molybdate or anisaldehyde staining reagents. Column chromatography was carried out on silica gel 60 (70–230 mesh). Custom Primer Support™ 200 Amino was purchased from GE Healthcare. Fmoc-L-Lys(Me_3_)-OH*Cl was purchased from Iris Biotech GmbH.

#### 4.1.2 9-(3′-Azido-3′-deoxy-β-D-arabinofuranosyl)-6-(1,2,4-triazol-4-yl)purine (**2**)

9-(3′-Azido-3′-deoxy-β-D-arabinofuranosyl)purine **1** (2.41 g, 8.25 mmol) and *N*,*N*-dimethylformamide azine dihydrochloride (6.20 g, 28.84 mmol) were dried over phosphorus pentoxide under high vaccum for 30 min and then suspended in 50 mL of dry pyridine. Chlorotriethylsilane (3.50 mL, 20.9 mmol) was added via syringe and the reaction mixture was refluxed for 24 h. All volatiles were evaporated under reduced pressure and the reddish-brown residue was dissolved in 200 mL of cold dichloromethane, washed with 200 mL of a cold 1:2 mixture of saturated sodium bicarbonate solution and brine and subsequently with 200 mL of a cold 1:2 mixture of 2 M HCl and brine. The organic phase was dried over Na_2_SO_4_ and evaporated. The resulting brown oil was dissolved in methanol (70 mL) and treated with 5.5 mL of 2 M aqueous HCl in portions until deprotection was deemed complete by TLC. All volatiles were evaporated in vacuo. Crude compound **2** can be purified by column chromatography on silica gel with 1–5% methanol in dichloromethane, or directly used in the next step. TLC (10% methanol in dichloromethane): *R_f_* = 0.22. ^1^H NMR (300 MHz, DMSO-*d*_6_): *δ* 3.65–3.79 (m, 2H, H(a)-C(5′), H(b)-C(5′)), 3.86–3.89 (m, 1H, H-C(4′)), 4.42 (t, *J* = 8.5 Hz, 1H, H-C(3′)), 4.68 (m, 1H, H-C(2′)), 5.32–5.35 (m, 1H, HO-C(5′)), 6.21 (d, *J* = 5.8 Hz, 1H, HO-C(2′)), 6.48 (d, *J* = 6.4 Hz, H-C(1′)), 8.94 (s, 1H, H-C(2) or H-C(8)), 8.99 (s, 1H, H-C(2) or H-C(8)), 9.65 (s, 2H, H-C(3)^triazole^ and H-C(5)^triazole^) ppm. ^13^C NMR (75 MHz, DMSO-*d*_6_): *δ* 60.00 (C(5′)), 63.56 (C(3′)), 74.53 (C(2′)), 80.39 (C(4′)), 83.06 (C(1′)), 140.90 (C(3)^triazole^ and C(5)^triazole^), 146.50 (C(2) or C(8)); 152.00 (C(2) or C(8)) ppm. ESI-MS (*m*/*z*): [M+H+Et_3_N]^+^ calcd for C_12_H_12_N_10_O_3_, 446.23; found 446.00.

#### 4.1.3 9-(3′-Azido-3′-deoxy-β-D-arabinofuranosyl)-6-(dimethylamino)purine (**3**)

Crude compound **2** from the previous reaction was dissolved in 40% aqueous dimethylamine (60 mL, 474 mmol) and 40 mL of pyridine and was stirred vigorously at room temperature for one hour, after which the substitution was judged complete by TLC. All volatiles were evaporated and the residue was purified by column chromatography on silica gel with 1–5% methanol in dichloromethane. Yield over two steps: 1.89 g of (**3**) as white solid (72%). TLC (10% methanol in dichloromethane): *R_f_* = 0.50. ^1^H NMR (300 MHz, DMSO-*d*_6_): *δ* 3.45 (s, 6H, C(6)-N(C*H*_3_)_2_), 3.61–3.72 (m, 2H, H(a)-C(5′), H(b)-C(5′)), 3.76–3.81 (m, 1H, H-C(4′)), 4.34 (t, *J* = 8.1 Hz, 1H, H-C(3′)), 4.45 (q, *J* = 6.7 Hz, 1H, H-C(2′)), 5.27 (m, 1H, HO-C(5′)), 6.07 (d, *J* = 5.8 Hz, 1H, HO-C(2′)), 6.28 (d, *J* = 6.4 Hz, 1H, H-C(1′)), 8.21 (s, 1H, H-C(2) or H-C(8)), 8.29 (s, 1H, H-C(2) or H-C(8)) ppm. ^13^C NMR (75 MHz, DMSO-*d*_6_): *δ* 37.91 (C(6)-N(*C*H_3_) _2_), 60.19 (C(5′)), 64.02 (C(3′)), 74.71 (C(2′)), 79.88 (C(4′)), 82.21 (C(1′)), 138.87 (C(2) or C(8)), 150.39 (C(2) or C(8)) ppm. ESI-MS (*m*/*z*): [M+H+Et_3_N]^+^ calcd for C_12_H_16_N_8_O_3_, 321.14; found 321.05.

#### 4.1.4 9-[3′-Azido-3′-deoxy-5′-*O*-(4,4′-dimethoxytrityl)-β-D-arabinofuranosyl]-6-(dimethylamino)purine (**4**)

Compound **3** (413 mg, 1.29 mmol) was dried over potassium hydroxide under high vacuum for 30 min and suspended in 5.5 mL of dry pyridine. 4,4′-Dimethoxytrityl chloride (524 mg, 1.55 mmol) was added in one portion and the yellow solution was stirred for 3.5 h. The reaction was quenched with 5 mL of methanol and volatiles were evaporated. The yellow residue was partitioned between 50 mL of ethyl acetate and 30 mL of 5% aqueous citric acid and the organic layer was washed with 5% aqueous citric acid, saturated sodium bicarbonate and brine (30 mL each). The organic phase was dried over MgSO_4_ and evaporated to provide a yellow foam. The product was purified by column chromatography on silica gel with 10% *n*-hexane in ethyl acetate, and subsequently, with neat ethyl acetate. Yield: 748 mg of **4** as white foam (93%). TLC (ethyl acetate): *R_f_* = 0.44. ^1^H NMR (300 MHz, DMSO-*d*_6_): *δ* 3.23–3.36 (m, 2H, H(a)-C(5′), H(b)-C(5′)), 3.46 (s, 6H, C(6)-N(C*H*_3_)_2_), 3.72 (s, 6H, 2 × O-C*H*_3_(DMT)), 3.92 (m, 1H, H-C(4′)), 4.46 (t, 1H, H-C(3′)), 4.54–4.58 (m, 1H, H-C(2′)), 6.14 (d, 1H, HO-C(2′)), 6.32 (d, 1H, H-C(1′)), 6.81–6.86 (t, 4H, H-C(ar)), 7.18–7.29 (m, 7H, H-C(ar)), 7.37 (d, 2H, H-C(ar)), 8.17 (s, 1H, H-C(2) or H-C(8)), 8.19 (s, 1H, H-C(2) or H-C(8)) ppm. ^13^C NMR (75 MHz, DMSO-*d*_6_): *δ* 37.88 (C(6)-N(*C*H_3_) _2_), 54.97 (2 × O-C*H*_3_(DMT)), 63.40 (C(5′)), 64.98 (C(3′)), 74.11 (C(2′)), 78.18 (C(4′)), 82.15 (C(1′)), 113.15 (C(ar)), 118.85, 126.70 (C(ar)), 127.65 (C(ar)), 127.79 (C(ar)), 129.63 (C(ar)), 129.71 (C(ar)), 135.28, 135.36, 139.05 (C(2) or C(8)), 144.68, 150.38, 151.84, 154.20 (C(2) or C(8)), 158.06, 158.10 ppm. ESI-MS (*m*/*z*): [M+H]^+^ calcd for C_33_H_34_N_8_O_5_, 623.27; found 623.22.

#### 4.1.5 9-[3′-Azido-3′-deoxy-2′-*O*-(trifluoromethanesulfonyl)-5′-*O*-(4,4′-dimethoxytrityl)-β-D-arabinofuranosyl]-6-(dimethylamino)purine (**4a**)

Compound **4** (650 mg, 1.04 mmol) and 4-(dimethylamino)pyridine (205 mg, 1.68 mmol) were weighed into an oven dried flask containing a magnetic stirring bar and were dried over potassium hydroxide under high vacuum for one hour. A rubber septum was then attached under argon and 17 mL of dry dichloromethane and *N*,*N*-diisopropylethylamine (490 μL, 2.81 mmol) were added. The flask was cooled in an icebath and trifluoromethanesulfonyl chloride (180 μL, 1.69 mmol) from a freshly opened ampule was slowly added. The reaction was allowed to proceed for 1 h at 0 °C and 2 h at room temperature, after which the starting material was no longer visible on TLC. The mixture was diluted with 75 mL of dichloromethane and washed with saturated sodium bicarbonate solution and brine (75 mL each), dried over Na_2_SO_4_ and evaporated to provide a brown foam. Compound **4a** was purified by silica gel column chromatography using 25–50% ethyl acetate in *n*-hexane. Yield: 658 mg of **4a** as white foam (84%). TLC (ethyl acetate/*n*-hexane 1:1): *R_f_* = 0.50. ^1^H NMR (300 MHz, DMSO-*d*_6_): *δ* 3.32–3.34 (m, 2H, H(a)-C(5′), H(b)-C(5′)), 3.46 (s, 6H, C(6)-N(C*H*_3_)_2_), 3.72 (s, 6H, 2 × O-C*H*_3_(DMT)), 4.19 (m, 1H, H-C(4′)), 5.02 (t, 1H, H-C(3′)), 5.67 (t, 1H, H-C(2′)), 6.26 (d, 1H, H-C(1′)), 6.80–6.85 (m, 4H, H-C(ar)), 7.20–7.28 (m, 7H, H-C(ar)), 7.35 (d, 2H, H-C(ar)), 8.18 (s, 1H, H-C(2) or H-C(8)), 8.35 (s, 1H, H-C(2) or H-C(8)) ppm. ESI-MS (*m*/*z*): [M+H]^+^ calcd for C_34_H_33_F_3_N_8_O_7_S, 755.22; found 755.19.

#### 4.1.6 3′-Azido-3′-deoxy-5′-*O*-(4,4′-dimethoxytrityl)-6-*N*-dimethyl-β-D-adenosine (**5**)

Triflate **4a** (300 mg, 0.397 mmol), 18-crown-6 (210 mg, 0.794 mmol) and potassium trifluoroacetate (333 mg, 2.19 mmol) were weighed into an oven dried flask containing a magnetic stirring bar and dried over potassium hydroxide under high vacuum for one hour. 15 mL of dry toluene and *N*,*N*-diisopropylethylamine (175 μL, 1.00 mmol) were added. The suspension was stirred for 6.5 h at 80 °C, then all volatiles were evaporated. The residue was partitioned between 50 mL of ethyl acetate and 50 mL of saturated sodium bicarbonate solution and the aqueous layer extracted with 30 mL of ethyl acetate. The organic layers were washed with 50 mL of brine, dried over MgSO_4_ and evaporated. Compound **5** was purified by silica gel gradient column chromatography using 50–0% *n*-hexane in ethyl acetate. Yield: 200 mg of **5** as white foam (81%). TLC (ethyl acetate/*n*-hexane 2:1): *R_f_* = 0.25. ^1^H NMR (300 MHz, DMSO-*d*_6_): *δ* 3.19–3.28 (m, 2H, H(a)-C(5′), H(b)-C(5′)), 3.45 (s, 6H, C(6)-N(C*H*_3_)_2_), 3.72 (s, 6H, 2 × O-C*H*_3_(DMT)), 4.12 (m, 1H, H-C(4′)), 4.42 (t, 1H, H-C(3′)), 5.07–5.12 (m, 1H, H-C(2′)), 5.97 (d, 1H, H-C(1′)), 6.33 (d, 1H, HO-C(2′)), 6.80–6.85 (m, 4H, H-C(ar)), 7.19–7.29 (m, 7H, H-C(ar)), 7.34 (d, 2H, H-C(ar)), 8.18 (s, 1H, H-C(2) or H-C(8)), 8.30 (s, 1H, H-C(2) or H-C(8)) ppm. ^13^C NMR (75 MHz, DMSO-*d*_6_): *δ* 37.93 (C(6)-N(*C*H_3_)_2_), 54.97 (2 × O-C*H*_3_(DMT)), 61.05 (C(3′)), 63.23 (C(5′)), 73.57 (C(2′)), 80.03 (C(4′)), 85.64, 88.16 (C(1′)), 113.13 (C(ar)), 119.68, 126.68 (C(ar)), 127.63 (C(ar)), 127.78 (C(ar)), 129.61 (C(ar)), 129.70 (C(ar)), 135.31, 135.41, 138.48 (C(2) or C(8)), 144.71, 150.03, 151.96, 154.27 (C(2) or C(8)), 158.04, 158.07 ppm. ESI-MS (*m*/*z*): [M+H]^+^ calcd for C_33_H_34_N_8_O_5_, 623.27; found 623.25.

#### 4.1.7 3′-Amino-3′-deoxy-5′-*O*-(4,4′-dimethoxytrityl)-*N*^6^,*N*^6^-dimethyl-β-D-adenosine (**5a**)

Compound **5** (300 mg, 0.482 mmol) was dissolved in 15 mL of ethyl acetate and palladium on activated charcoal (35 mg, 10% Pd, 33 μmol Pd) was added. The flask was equipped with a rubber septum and flushed with argon. Hydrogen was bubbled through the stirred suspension for 15 min and then the reaction was held under positive hydrogen pressure for 22 h. The suspension was filtered through a pad of celite and the solvent was evaporated to a white foam of high purity. Product **5a** was purified by column chromatography on silica gel with 1–9% methanol in dichloromethane. Yield: 264 mg of **5a** as white foam (92%). TLC (10% methanol in dichloromethane): *R_f_* = 0.38. ^1^H NMR (300 MHz, DMSO-*d*_6_): *δ* 1.58 (s, 2H, NH_2_-C(3′)), 3.18–3.27 (m, 2H, H(a)-C(5′), H(b)-C(5′)), 3.45 (s, 6H, C(6)-N(C*H*_3_)_2_), 3.60–3.65 (m, 1H, H-C(3′)), 3.72 (s, 6H, 2 × O-C*H*_3_(DMT)), 3.83–3.85 (m, 1H, H-C(4′)), 4.39 (d, 1H, H-C(2′)), 5.85 (s, 1H, HO-C(2′)), 6.00 (d, 1H, H-C(1′)), 6.78–6.83 (m, 4H, H-C(ar)), 7.16–7.26 (m, 7H, H-C(ar)), 7.35 (d, 2H, H-C(ar)), 8.23 (s, 1H, H-C(2) or H-C(8)), 8.24 (s, 1H, H-C(2) or H-C(8)) ppm. ^13^C NMR (75 MHz, DMSO-*d*_6_): *δ* 37.88 (C(6)-N(*C*H_3_)_2_), 53.35 (C(3′)), 54.94 (2 × O-CH_3_ (DMT)), 63.78 (C(5′)), 74.27 (C(2′)), 83.25 (C(4′)), 85.44, 89.31 (C(1′)), 113.04, 119.63, 126.55, 127.70, 129.66, 129.74, 135.59, 135.65 (C(ar)), 137.86 (C(2) or C(8)), 144.94, 149.80, 151.90 (C(2) or C(8)), 154.24, 157.94, 157.98 ppm. ESI-MS (*m*/*z*): [M+H]^+^ calcd for C_33_H_36_N_6_O_5_, 597.28; found 596.89.

#### 4.1.8 3′-{[*N*-(9-Fluorenyl)methoxycarbonyl-glycyl]amino}-3′-deoxy-5′-*O*-(4,4′-dimethoxytrityl)-*N*^6^,*N*^6^-dimethyl-β-D-adenosine (**6**)

Compound **5a** (165 mg, 0.276 mmol) and *N*-(9-fluorenyl)methoxycarbonyl glycine pentafluorophenyl ester (165 mg, 0.356 mmol) were dried under high vacuum for 30 min. 2 mL of dry DMF was then added and the solution was stirred for 5.5 h at room temperature. The reaction mixture was diluted with 30 mL of dichloromethane, washed with saturated sodium bicarbonate solution and brine, dried over Na_2_SO_4_ and evaporated. Compound **6** was purified by silica gel column chromatography using 1–9% methanol in dichloromethane as the eluent. Yield: 230 mg off-white solid (95%). TLC (7% methanol in dichloromethane): *R_f_* = 0.27. ^1^H NMR (300 MHz, DMSO-*d*_6_): *δ* 3.24 (bd, 2H, H(a)-C(5′), H(b)-C(5′)), 3.46 (br s, 6H, C(6)-N(C*H*_3_)_2_), 3.70 (s, 8H, 2 × O-C*H*_3_ (DMT), CH_2_ (α, Gly)), 4.14 (m, 1H, H-C(4′)), 4.20–4.31 (m, 3H, H-C(9, Fmoc)), OCH_2_ (Fmoc)), 4.64 (m, 1H, H-C(2′)), 4.71–4.79 (m, 1H, H-C(3′)), 6.01 (bd, 1H, H-C(1′)), 6.10 (d, *J* = 4.6 Hz, 1H, HO-C(2′)), 6.79–6.84 (m, 4H, H-C(ar, DMT)), 7.19–7.34 (m, 12H, H-C(ar)), 7.38–7.43 (m, 2H, H-C(ar)), 7.53 (t, *J* = 6.0 Hz, 1H, NH(Gly)), 7.70–7.72 (m, 2H, H-C(ar)), 7.87–7.90 (m, 2H, H-C(ar)), 7.94 (d, *J* = 8.0 Hz, 1H, NH-C(3′)), 8.21 (s, 1H, H-C(2) or H-C(8)), 8.27 (s, 1H, H-C(2) or H-C(8)) ppm. ^13^C NMR (75 MHz, DMSO-*d*_6_): *δ* 37.89 (C(6)-N(*C*H_3_)_2_), 43.30 (C(α, Gly)), 46.61 (C(9, Fmoc)), 50.60 (C(3′)), 54.93 (2 × O-CH_3_(DMT)), 62.72 (C(5′)), 65.71 (OCH_2_(Fmoc)), 72.60 (C(2′)), 80.82 (C(4′)), 85.55, 89.62 (C(1′), 113.10, 119.75, 120.08, 125.21, 126.58, 127.06, 127.60, 127.68, 127.74, 129.62, 129.73, 135.35, 135.46, 137.68 (C(2) or C(8)), 140.6939, 143.80, 144.75, 149.72 (C(ar)), 151.93 (C(2) or C(8)), 154.26, 156.42, 157.96, 158.00, 169.22 ppm.

#### 4.1.9 3′-{[*N*-(9-Fluorenyl)methoxycarbonyl-glycyl]amino}-3′-deoxy-5′-*O*-(4,4′-dimethoxytrityl)-*N*^6^,*N*^6^-dimethyl-2′-*O*-[1,6-dioxo-6-(pentafluorophenyloxy)hexyl]-β-D-adenosine (**7**)

Compound **6** (289 mg, 0.331 mmol), 4-(dimethylamino)pyridine (40 mg, 0.331 mmol) and bis(pentafluorophenyl) adipate (506 mg, 1.06 mmol) were dried over phosphorus pentoxide under high vacuum for 30 min. Dry pyridine and dry DMF (5 mL each) was added and the solution was stirred for 4 h at room temperature, after which the starting material was no longer visible on TLC. All volatiles were evaporated under high vacuum and compound **7** was purified by silica gel column chromatography with 0–30% acetone in dichloromethane. Yield: 198 mg white foam (51%). TLC (25% acetone in dichloromethane): *R_f_* = 0.41. ^1^H NMR (300 MHz, DMSO-*d*_6_): *δ* 1.62 (br s, 4H, 2 × CH_2_(adipate)), 2.75 (m, 2H, CH_2_(adipate)), 3.21 (m, 2H, HC(5′)), 3.46 (br s, 6H, C(6)-N(C*H*_3_)_2_), 3.65 (m, 2H, CH_2_(α, Gly)), 3.70 (s, 2 × O-C*H*_3_(DMT)), 4.21–4.29 (m, 4H, H-C(4′), H-C(9, Fmoc), OCH_2_(Fmoc)), 5.09 (m, 1H, H-C(3′)), 5.83–5.87 (m, 2H, H-C(2′)), 6.20 (d, *J* = 3.1 Hz, 1H, H-C(1′)), 6.78–6.82 (m, 4H, H-C(ar)), 7.17–7.41 (m, 14H, H-C(ar)), 7.56 (t, *J* = 6.1 Hz, 1H, NH(Gly)), 7.69 (d, *J* = 7.2 Hz, 2H, H-C(ar)), 7.87 (d, *J* = , 7.3 Hz, 2H, H-C(ar)), 8.17 (m, 1H, NH(C3′)), 8.20 (s, 1H, H-C(2) or H-C(8)), 8.28 (s, 1H, H-C(2) or H-C(8)) ppm. ESI-MS (*m*/*z*): [M+Na]^+^ calcd for C_62_H_56_F_5_N_7_O_11_, 1192.39; found 1192.30.

#### 4.1.10 DMTO-m^6^_2_A-3′-NH-(Fmoc-Gly) solid support (**8**)

Active ester **7** (187 mg, 0.160 mmol) and amino-functionalized support (GE Healthcare, Custom Primer Support™ 200 Amino, 560 mg) were weighed into a 10 mL flask and dried under high vacuum. The flask was flushed with argon and equipped with a rubber septum. Dry pyridine (26 μL, 0.320 mmol) and dry DMF (3.5 mL) were added and the reaction vessel was agitated for one day. The solid support was filtered and washed with DMF, methanol and dichloromethane (twice 10 mL each) and dried. The beads were capped with a mixture of 10 mL 0.2 M phenoxyacetic anhydride in dry THF and 10 mL of 0.2 M 1-methyl imidazole and 0.2 M 2,4,6-trimethylpyridine in dry THF for 10 min at room temperature. The suspension was filtered and the support was washed with acetonitrile, methanol, and dichloromethane (50 mL each) and dried under vacuum. The very first filtrate, containing residual active ester **7**, was used to treat another 400 mg of amino-functionalized support for 3.5 days. Loading of the first batch of support **8** was 45 μmol/g; loading of the second batch was 71 μmol/g.

#### 4.1.11 3′-{[*N*-(9-Fluorenyl)-methoxycarbonyl-*N*^6^,*N*^6^,*N*^6^-(trimethyl)-L-lysyl]amino}-3′-deoxy-5′-*O*-(4,4′-dimethoxytrityl)-*N*^6^,*N*^6^-dimethyl-β-D-adenosine (**9**)

Fmoc protected *N*^6^,*N*^6^,*N*^6^-trimethyl-L-lysine chloride (90 mg, 0.20 mmol), HBTU (64 mg, 0.17 mmol), HOBt monohydrate (26 mg, 0.17 mmol) and *N*,*N*-diisopropylethylamine (35 μL, 0.20 mmol) were dissolved in 3.0 mL of DMF. After 5 min compound **5a** (78 mg, 0.13 mmol) was added and the solution stirred for 12 h. All volatiles were evaporated and the residue partitioned between 50 mL of dichloromethane and 5% aqueous citric acid. The organic layer was washed with saturated sodium bicarbonate solution and brine (50 mL each). Due to perseverant emulsions, the aqueous phases were re-extracted with 30 mL of dichloromethane. The organic phases were combined and dried over molecular sieves. After evaporation of the solvent, a yellow foam was obtained. The crude product was purified by silica gel column chromatography using a gradient from 5% to 10% methanol in dichloromethane. Yield: 36 mg (27% calculated as chloride salt) of compound **9** as a greyish solid. TLC (10% methanol in dichloromethane): *R_f_* = 0.10. ^1^H NMR (300 MHz, DMSO-*d*_6_): *δ* 1.24 (m, 2H, CH_2_(γ, Lys)), 1.50–1.63 (m, 4H, CH_2_(β, Lys), CH_2_(δ, Lys)), 3.02 (s, 9H, (CH_3_)_3_N^+^), 3.16–3.25 (m, 4H, H(a)-C(5′), H(b)-C(5′), CH_2_(ε, Lys)), 3.45 (br s, 6H, C(6)-N(C*H*_3_)_2_), 3.72 (s, 6H, 2 × O-C*H*_3_(DMT)), 4.12–4.33 (m, 5H, H-C(4′), H-C(9, Fmoc), OCH_2_(Fmoc), CH(α, Lys)), 4.66 (m, 1H, H-C(2′)), 4.72–7.79 (m, 1H, H-C(3′)), 6.03 (m, 1H, HO-C(2′)), 6.18 (d, *J* = 4.5 Hz, 1H, H-C(1′)), 6.79–6.83(m, 4H, H(ar)), 7.18–7.27 (m, 7H, H(ar)), 7.30–7.44 (m, 6H, H(ar)), 7.52 (d, *J* = 8.5 Hz, 1H, NH(Lys)), 7.69–7.73 (m, 2H, H(ar)), 7.89 (d, *J* = 7.4 Hz, 2H, H(ar)), 8.01 (d, *J* = 8.3 Hz, 1H, NH(C3′)), 8.21 (s, 1H, C(2) or C(8)), 8.27 (s, 1H, C(2) or C(8)) ppm. ESI-MS (*m*/*z*): [M^+^] calcd for C_57_H_65_N_8_O_8_, 989.49; found 989.58.

#### 4.1.12 DMTO-m^6^_2_A-3′-NH-(Fmoc-Lys(CH_3_)_3_) solid support (**10**)

Compound **9** (30 mg, 0.029 mmol), 4-dimethylaminopyridine (4 mg, 0.03 mmol) and bis(pentafluorophenyl) adipate (30 mg, 0.06 mmol) were dried in high vacuum for 2 h and dissolved in 1.5 mL of dry DMF and 1.0 ml of dry pyridine. After 2 h TLC indicated complete conversion to intermediate **9a** and amino-functionalized support (GE Healthcare, Custom Primer Support™ 200 Amino, 150 mg) was added. The suspension was agitated for one day at room temperature and then filtrated. The solid support was washed with DMF, methanol, dichloromethane (2 × 10 mL each) and dried in high vacuum. Capping was performed with a mixture of 10 mL 0.2 M phenoxyacetic anhydride in dry THF and 10 mL of 0.2 M 1-methyl imidazole and 0.2 M 2,4,6-trimethylpyridine in dry THF for 20 min at room temperature. The beads were filtered and washed with acetonitrile, methanol and dichloromethane (50 mL each). The very first filtrate, containing residual active ester, was used to treat another 100 mg of amino-functionalized support for 4 days. Loading of the first batch of support **10** was 18 μmol/g; loading of the second batch was 11 μmol/g.

### 4.2 Solid-phase peptide and oligonucleotide synthesis, deprotection and purification of RNA-amino acid conjugates

#### 4.2.1 Manual solid-phase peptide synthesis on the amino acyl charged solid support **8**

The solid support **8** (4 μmol loaded conjugate) was transferred to a fritted syringe. After each reaction step the supernatant reagent solutions were pushed through the filter and the solid support was washed with twice 3 mL DMF. After initial soaking with DMF, Fmoc protecting groups were removed by treating twice with 20% piperidine in DMF (1.5 mL, 8 min; 1.5 mL 12 min). Coupling was achieved by agitation of the beads with a premixed solution of 0.4 M Fmoc protected amino acid in 500 μL of DMF, 750 μL activator solution in DMF (0.6 M *O*-(benzotriazol-1-yl)-*N*,*N*,*N*′,*N*′-tetramethyluronium hexafluorophosphate, 0.6 M 1-hydroxybenzotriazole) and 140 μL of *N*,*N*-diisopropylethylamine at room temperature for one hour. This coupling step was repeated once to ensure complete conversion. Finally, the support was washed thoroughly with acetonitrile, dried in high vacuum and supplied to solid-phase oligonucleotide synthesis.

#### 4.2.2 Solid-phase oligonucleotide synthesis on the amino acyl charged solid supports **8** and **10**

All oligonucleotides were synthesized on a 1.0 μmol scale using an *Applied Biosystems* ABI 392 following standard synthesis protocols. Detritylation (2 min): 4% (v/v) dichloroacetic acid in 1,2-dichloroethane. Coupling (3 min): 120 μL 0.1 M phosphoramidite in acetonitrile plus 360 μL 0.30 M 5-(benzylthio)-1*H*-tetrazole in acetonitrile as activator. Capping (2 × 0.5 min): 1:1 (v/v) Cap A/Cap B, Cap A: 0.2 M phenoxyacetic anhydride in dry THF, Cap B: 0.2 M 1-methyl imidazole and 0.2 M 2,4,6-trimethylpyridine in dry THF. Oxidation (1 min): 20 mM iodine in 7/2/1 (v/v/v) THF / pyridine / water. Phosphoramidite and activator solutions were dried over activated molecular sieves (3 Å) overnight. All sequences were synthesized trityl-off.

#### 4.2.3 Deprotection of RNA-amino acid conjugates

The solid support was treated with 20 mL of 12% (v/v) diethylamine in acetonitrile over 15 min, washed with 20 mL of acetonitrile and dried in high vacuum. The beads were transferred into a screw-capped *Eppendorf* tube and 1.5 mL of a 3:1 mixture of 30% aqueous ammonia and ethanol was added and the reaction proceeded at 45 °C for 4 h with occasional shaking. The suspension was filtered and all volatiles evaporated. The residue was dissolved in 1.3 mL of 1 M tetrabutylammonium fluoride trihydrate in THF and kept at 37 °C for 12 h. The reaction was quenched by addition of 1.3 mL of triethylammonium bicarbonate buffer (1 M, pH 7.4) and the organic solvent evaporated. The solution was diluted with triethylammonium bicarbonate buffer (100 mM, pH 7.4) to approximately 2 mL, applied onto an equilibrated C18 SepPak®Plus cartridge (*Waters*), washed with water and the aminoacylated RNA eluted with water/acetonitrile (1/1, v/v). The dried crude product was redissolved in 1.0 mL of water.

#### 4.2.4 Analysis and purification of RNA-amino acid conjugates

Analysis of crude products was performed by anion-exchange HPLC on a *Dionex* DNAPac®PA-100 column (4 × 250 mm) at 60 °C. Flow rate: 1 mL/min; eluant A: 25 mM Tris·HCl (pH 8.0), 6 M urea; eluant B: 25 mM Tris·HCl (pH 8.0), 6 M urea, 500 mM NaClO_4_; gradient: 0–40% B in A within 25 min; UV-detection at 260 nm. Crude products were purified on a semi-preparative *Dionex* DNAPac®PA-100 column (9x250 mm) at 60 °C. Flow rate: 2 mL/min; gradient: 3–10% B in A within 18 min for tetranucleotides, 13–23% B in A within 18 min for hexanucleotides; UV-detection at 260 nm. Fractions containing the oligonucleotide were diluted with en equal volume of triethylammonium bicarbonate buffer (100 mM, pH 7.4) and loaded on an equilibrated C18 SepPak®Plus cartridge (*Waters)*, washed with water and eluted with water/acetonitrile (1/1, v/v). Purified fractions were evaporated and redissolved in 1.0 mL water. The RNA yield was determined as units of optical density at 260 nm by UV spectroscopy (*Implen* NanoPhotometer) at room temperature. The product quality and purity was verified by anion-exchange chromatography on an analytical column (*vide supra*).

#### 4.2.5 LC-ESI mass spectrometry of RNA-peptide conjugates

All analyses were performed on a Finnigan LCQ Advantage MAX ion trap instrumentation (*Thermo Fisher Scientific*) connected to an Amersham Ettan micro LC system (*GE Healthcare*). RNA-amino acid conjugates were analyzed in the negative-ion mode with a potential of −4 kV applied to the spray needle. LC: sample: 200 pmol lyophilized conjugate dissolved in 30 μL. Column: XTerra®MS, C18 2.5 μm, 1.0 × 50 mm at 21 °C; flow rate: 30 μL/min; eluant A: 8.6 mM triethylamine, 100 mM 1,1,1,3,3,3-hexafluoroisopropanol in water (pH 8.0); eluant B: methanol; gradient: 0–100% B in A within 20 min; UV-detection at 254 nm. Prior each injection, column equilibration was performed by eluting buffer A for 30 min at a flow rate of 30 μL/min.

## Supplementary data

Supplementary data ^1^H and ^13^C NMR spectra for compounds **2** to **9**.
