# Supplementary material for: Synthesis of aminoacylated N6,N6-dimethyladenosine solid support for efficient access to hydrolysis-resistant 3′-charged tRNA mimics
Source: Bioorg Med Chem. 2014 Dec 15;22(24):6989–95. doi: 10.1016/j.bmc.2014.09.054 (PMC4270447; doi:10.1016/j.bmc.2014.09.054)

SUPPORTING INFORMATION TO

Synthesis of aminoacylated *N*^{6}, *N*^{6}-dimethyladenosine solid support for efficient access to hydrolysis-resistant 3'-charged tRNA mimics

*Sandro Neuner and Ronald Micura**

Institute of Organic Chemistry and Center for Molecular Biosciences CMBI, University of Innsbruck, Innrain 80-82, 6020 Innsbruck, Austria

---

CONTENTS

^{1}H and ^{13}C NMR spectra of compounds 2 to 7, and 9

compound **2**	**S2**
compound **3**	**S3**
compound **4**	**S4**
compound **4a**	**S5**
compound **5**	**S6**
compound **5a**	**S7**
compound **6**	**S8**
compound **7**	**S9**
compound **9**	**S10**

9-(3'-Azido-3'-deoxy-β-D-arabinofuranosyl)-6-(1,2,4-triazol-4-yl)purine (2)

^{1}H NMR (300 MHz, DMSO-d_6) of compound **2**:

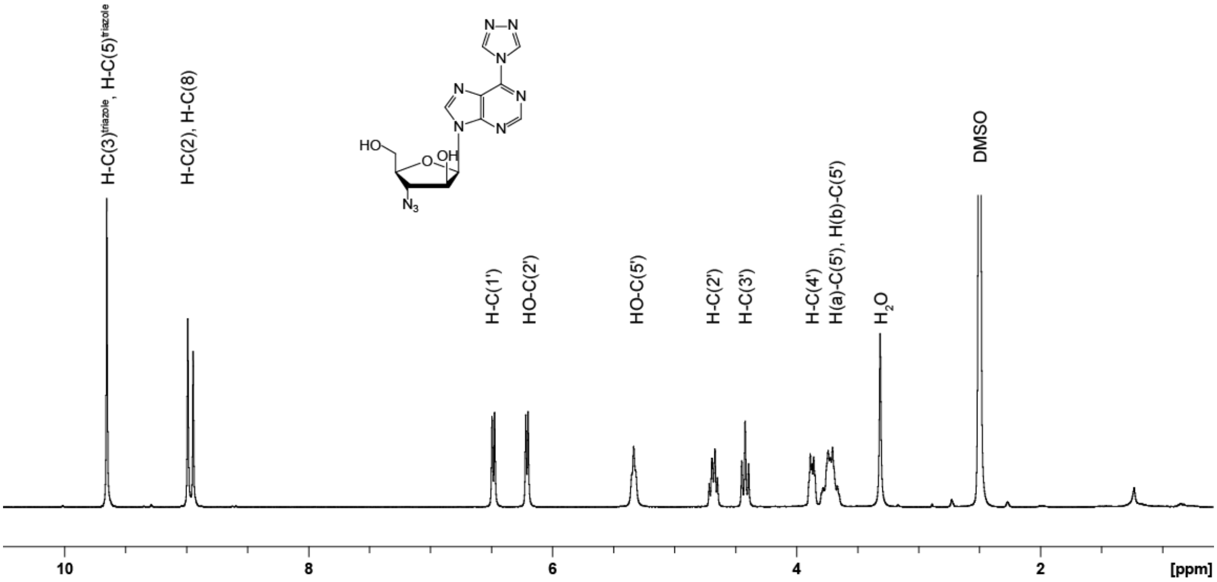

^{13}C NMR (75 MHz, DMSO-d_6) of compound **2**:

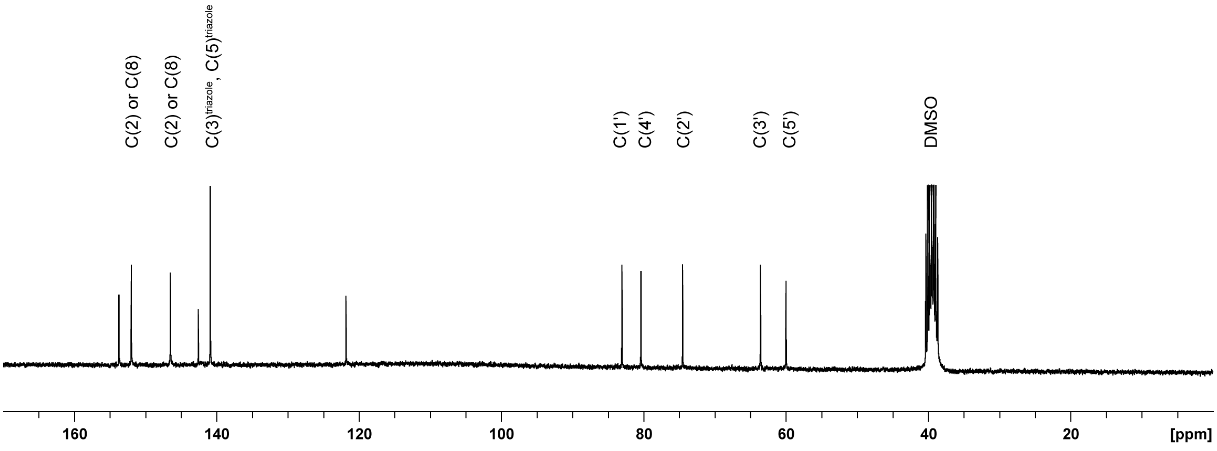

9-(3'-Azido-3'-deoxy-*β*-D-arabinofuranosyl)-6-(dimethylamino)purine (3)

^{1}H NMR (300 MHz, DMSO-d_6) of compound **3**:

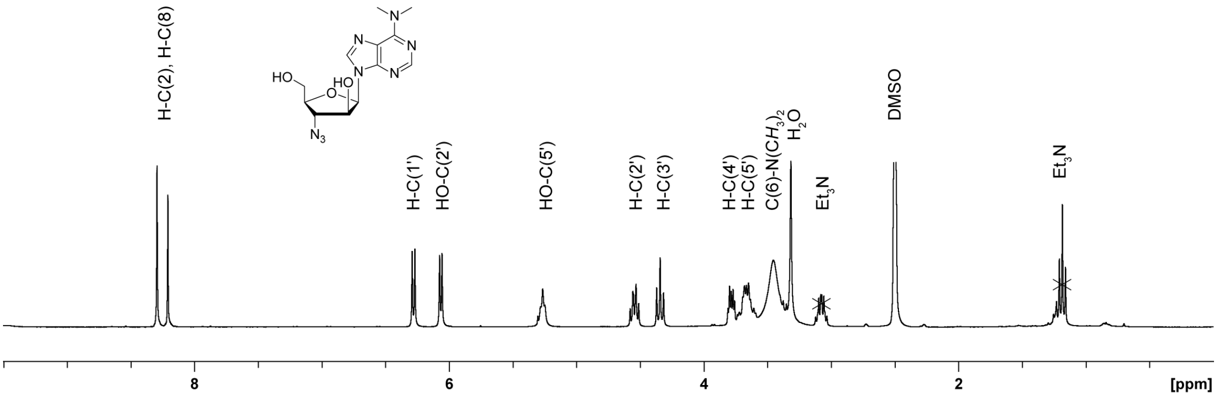

^{13}C NMR (75 MHz, DMSO-d_6) of compound **3**:

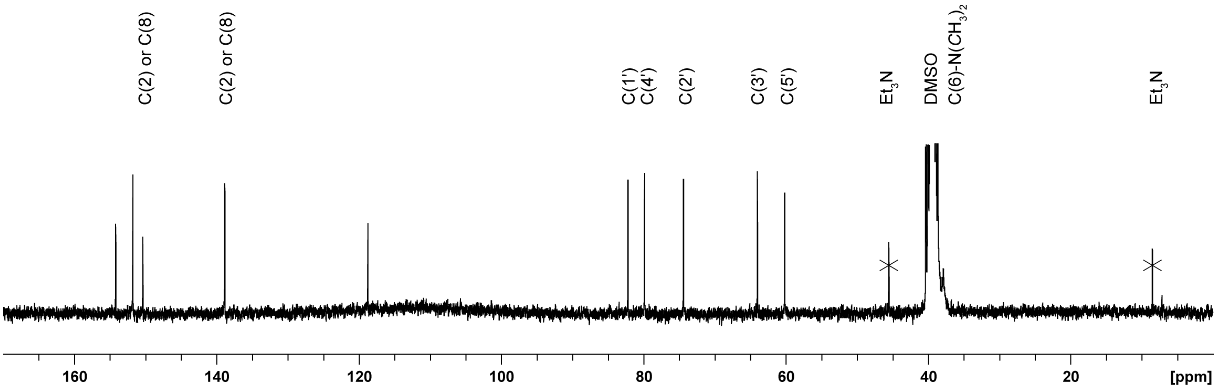

9-[3'-Azido-3'-deoxy-5'-*O*-(4,4'-dimethoxytrityl)-*β*-D-arabinofuranosyl]-6-(dimethylamino)purine (4)

^{1}H NMR (300 MHz, DMSO-d_6) of compound **4**:

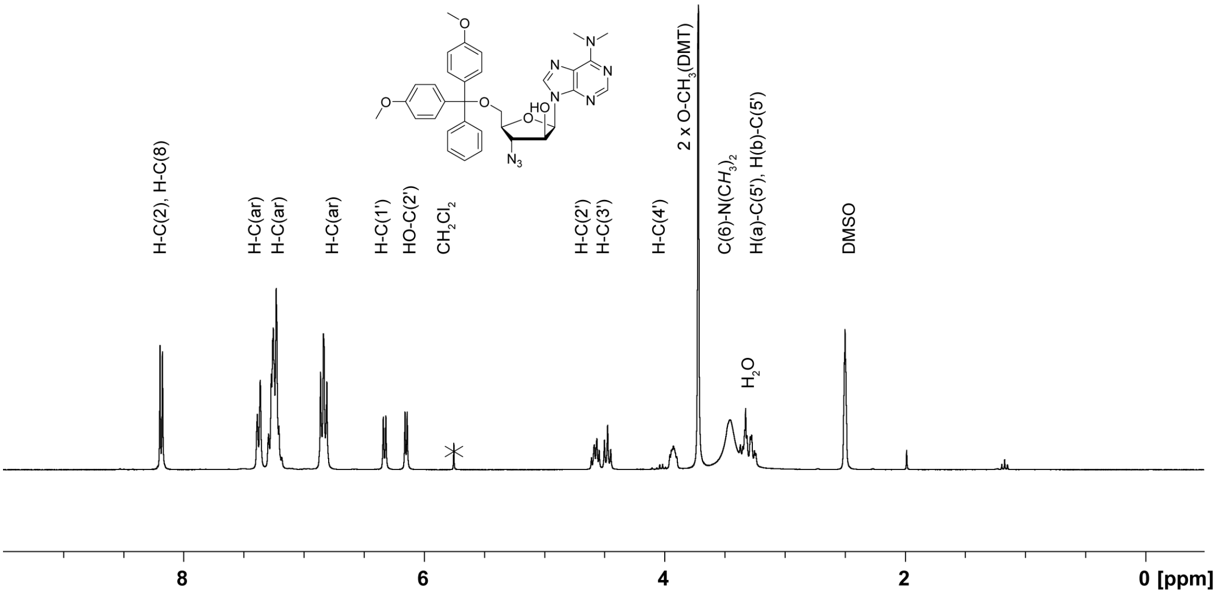

^{13}C NMR (75 MHz, DMSO-d_6) of compound **4**:

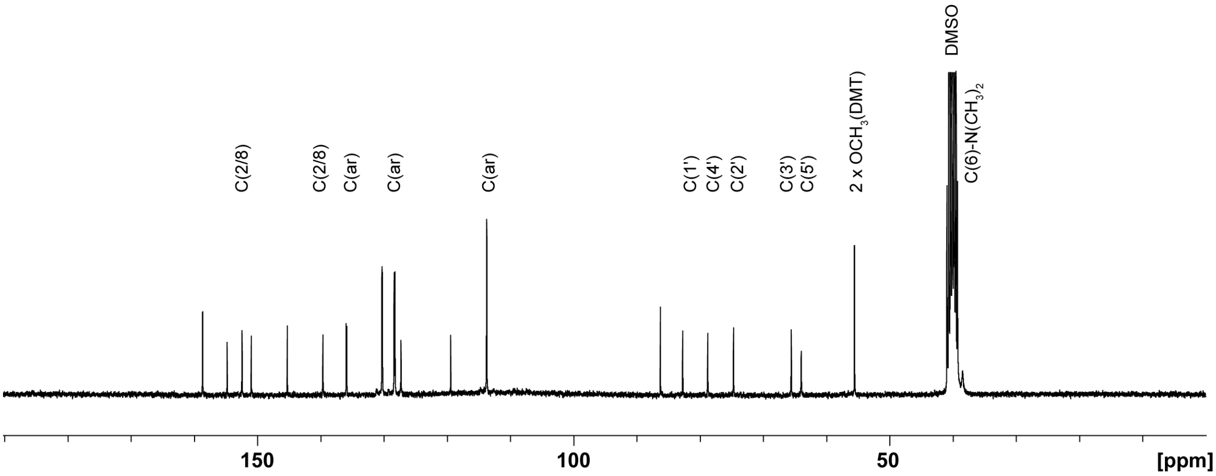

9-[3'-Azido-3'-deoxy-2'-*O*-(trifluoromethanesulfonyl)-5'-*O*-(4,4'-dimethoxytrityl)-*β*-D-arabinofuranosyl]-6-(dimethylamino)purine (4a)

^{1}H NMR (300 MHz, DMSO-d$_6$) of compound **4a**:

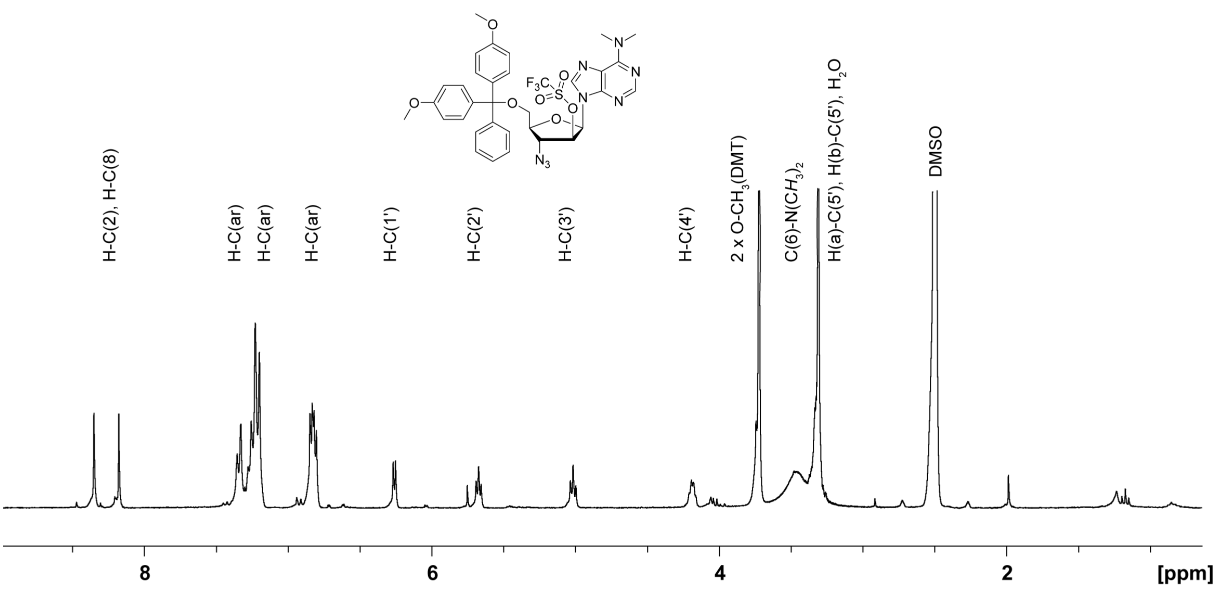

3'-Azido-3'-deoxy-5'-*O*-(4,4'-dimethoxytrityl)-*N*^{6},*N*^{6}-dimethyl-*β*-D-adenosine (5)

^{1}H NMR (300 MHz, DMSO-d$_6$) of compound **5**:

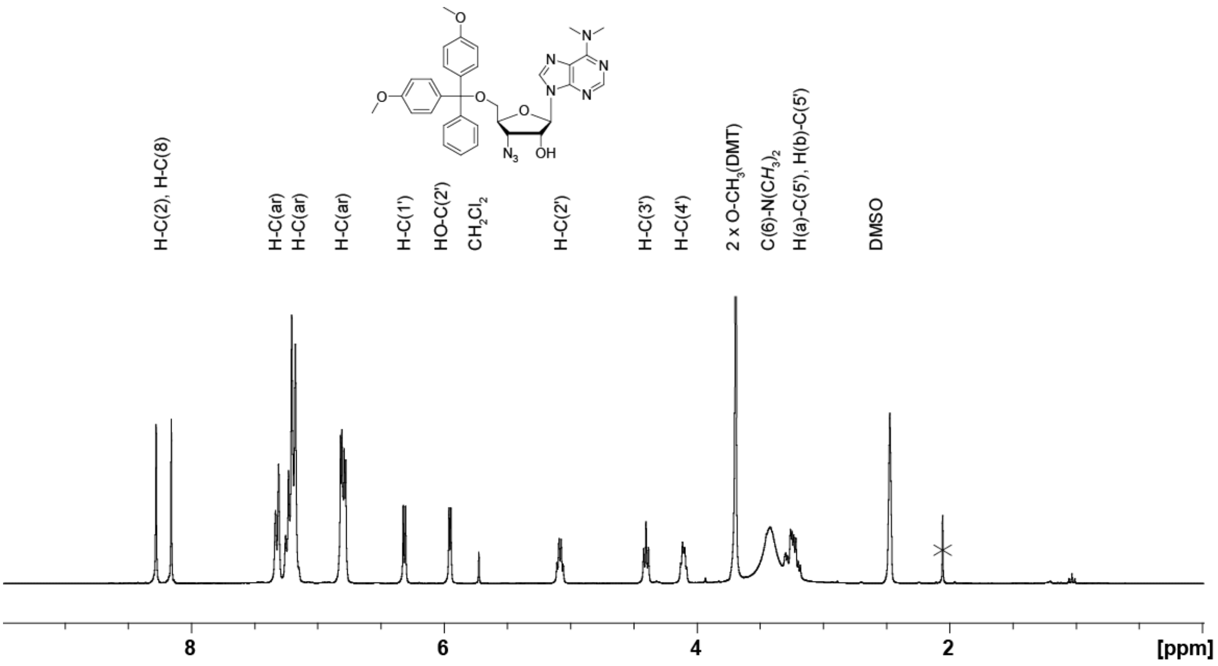

^{13}C NMR (75 MHz, DMSO-d$_6$) of compound **5**:

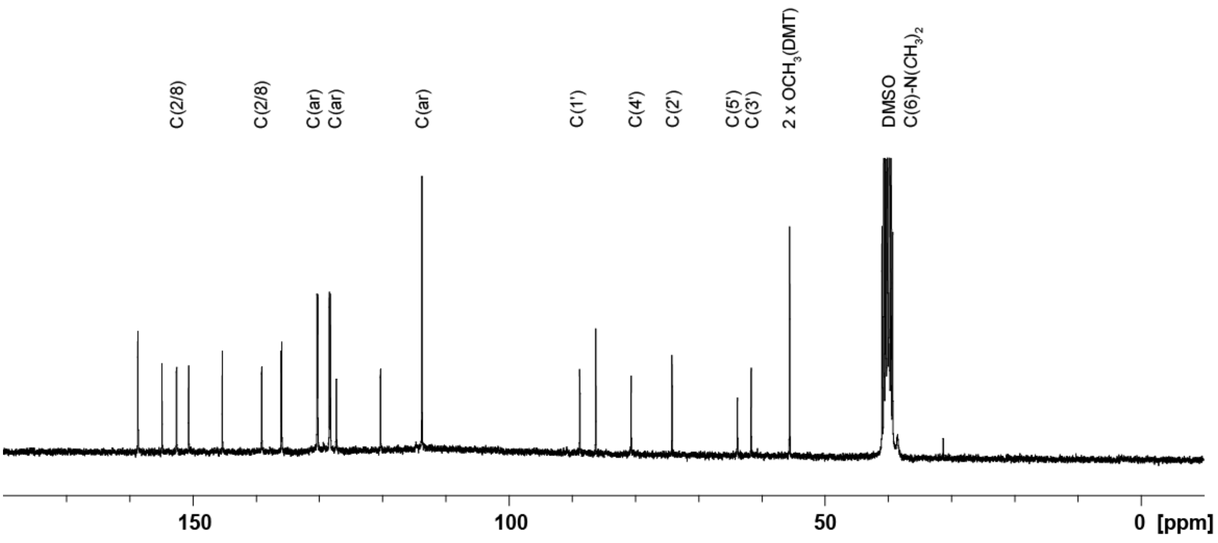

3'-Amino-3'-deoxy-5'-*O*-(4,4'-dimethoxytrityl)-*N*^{6},*N*^{6}-dimethyl-*β*-D-adenosine (5a)

^{1}H NMR (300 MHz, DMSO-d$_6$) of compound **5a**:

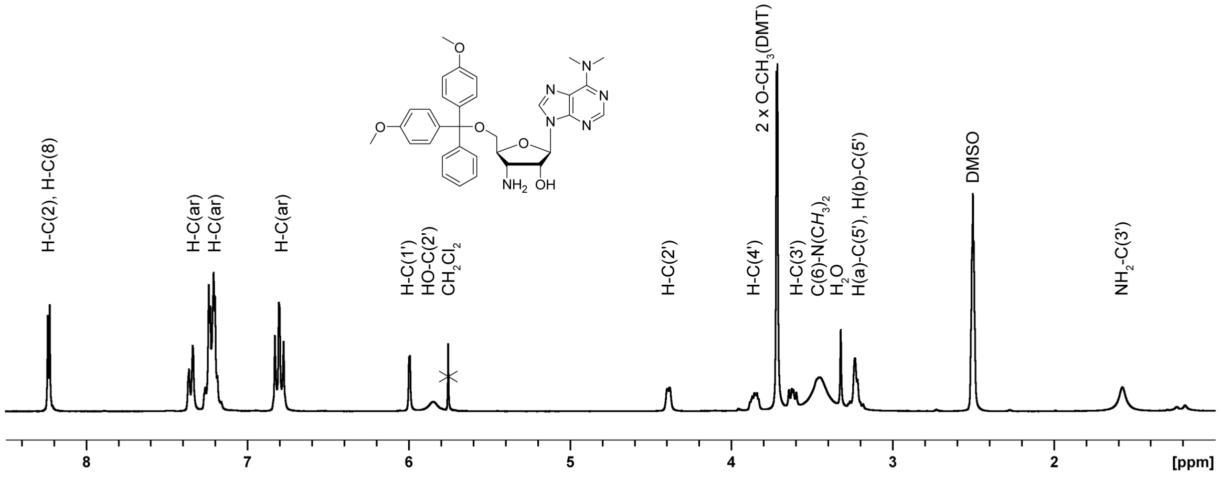

^{13}C NMR (75 MHz, DMSO-d$_6$) of compound **5a**:

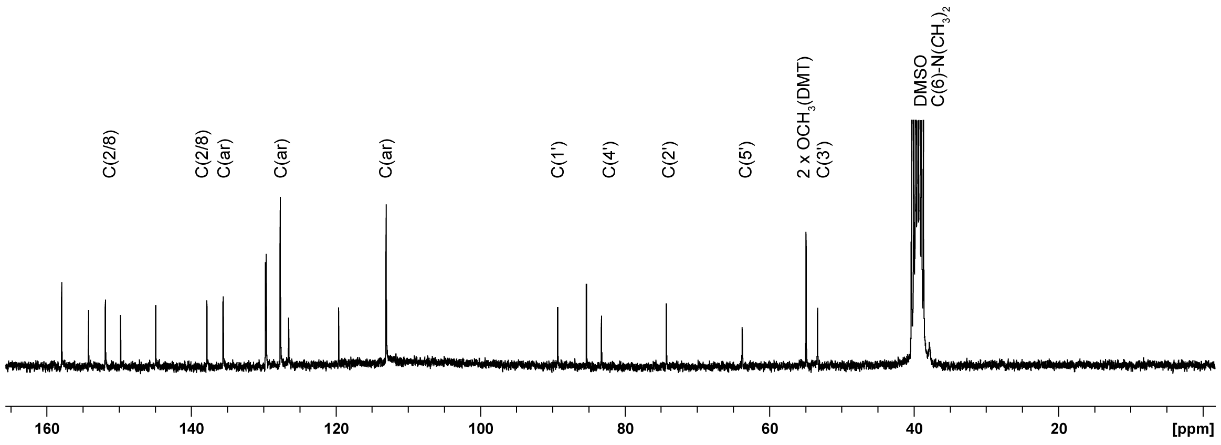

3'-{[*N*-(9-fluorenyl)methoxycarbonyl-glycyl]amino}-3'-deoxy-5'-*O*-(4,4'-dimethoxytrityl)-*N*^{6},*N*^{6}-dimethyl-*β*-D-adenosine (6)

^{1}**H NMR** (300 MHz, DMSO-d$_6$) of compound **6**:

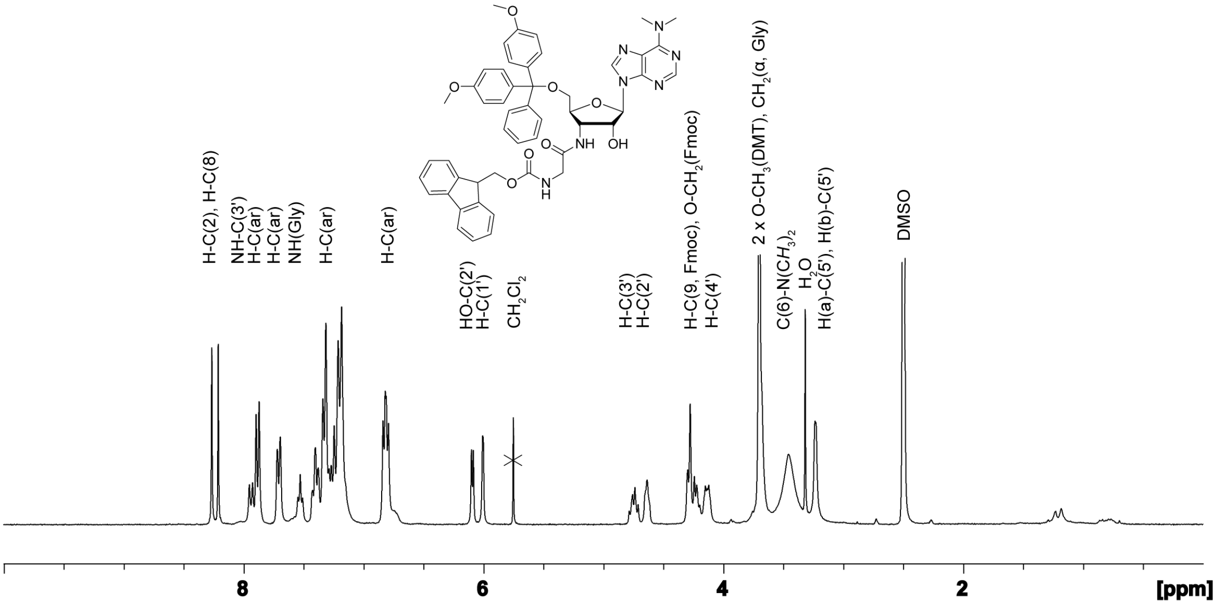

^{13}**C NMR** (75 MHz, DMSO-d$_6$) of compound **6**:

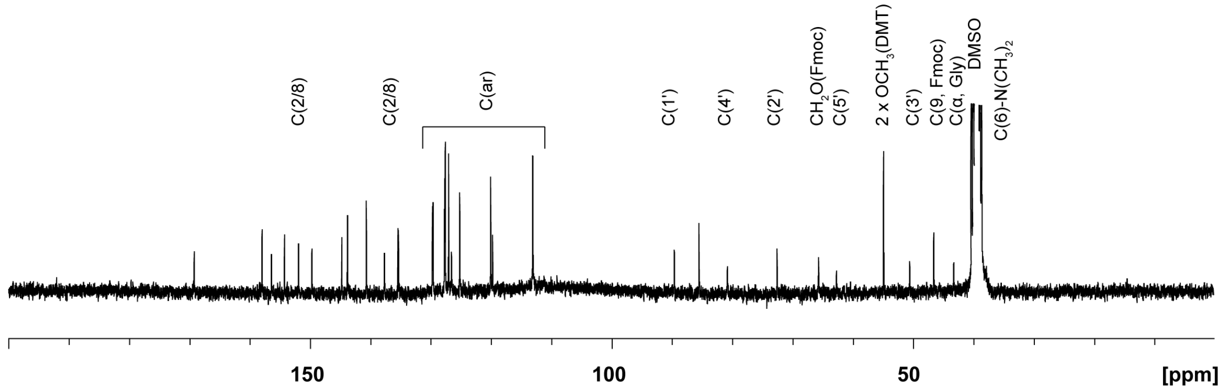

3'-{[*N*-(9-fluorenyl)methoxycarbonyl-glycyl]amino}-3'-deoxy-5'-*O*-(4,4'-dimethoxytrityl)-*N*^{6},*N*^{6}-dimethyl-2'-*O*-[1,6-dioxo-6-(pentafluorophenyloxy)hexyl]-*β*-D-adenosine (7)

^{1}H NMR (300 MHz, DMSO-d_6) of compound **7**:

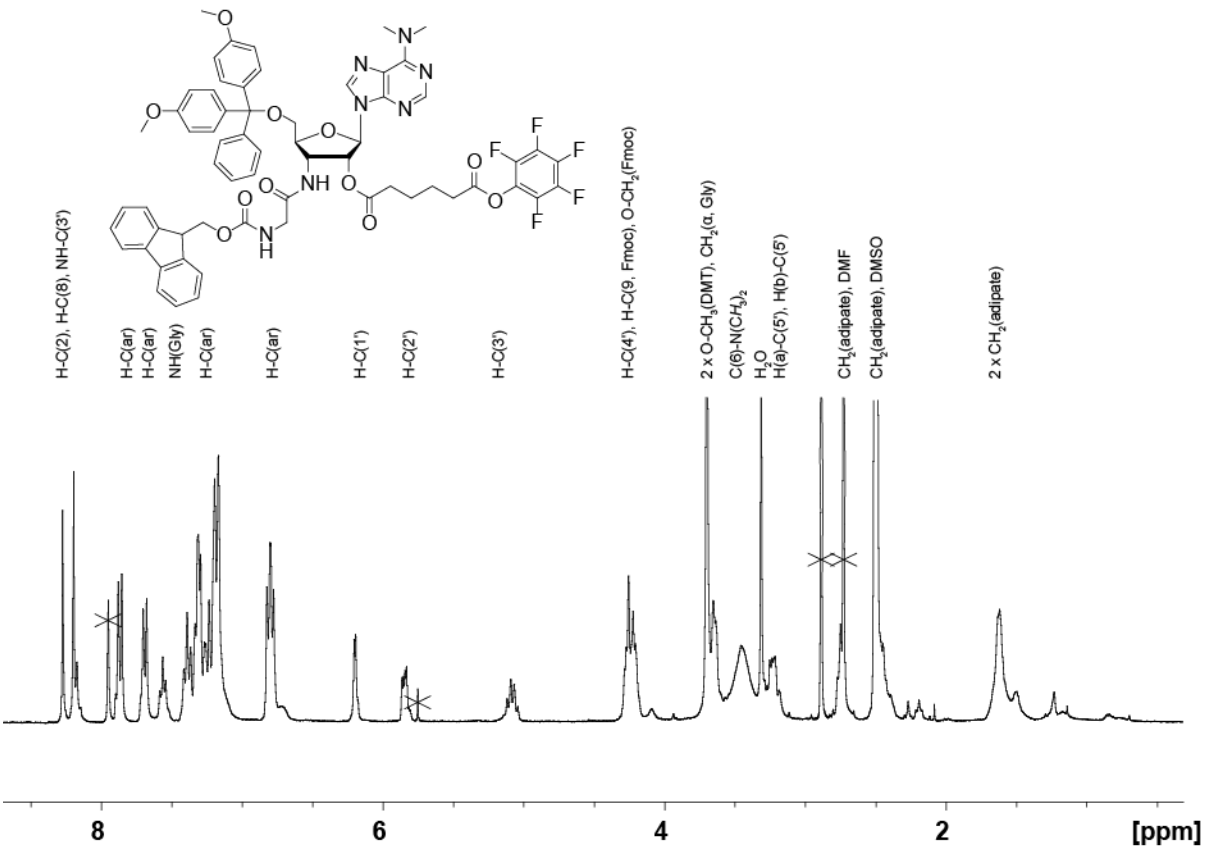

3'-{[*N*-(9-fluorenyl)-methoxycarbonyl-*N*^{6},*N*^{6},*N*^{6}-(trimethyl)-L-lysyl]amino}-3'-deoxy-5'-*O*-(4,4'-dimethoxytrityl)-*N*^{6},*N*^{6}-dimethyl-*β*-D-adenosine (9)

^{1}H NMR (300 MHz, DMSO-d$_6$) of compound **9**:

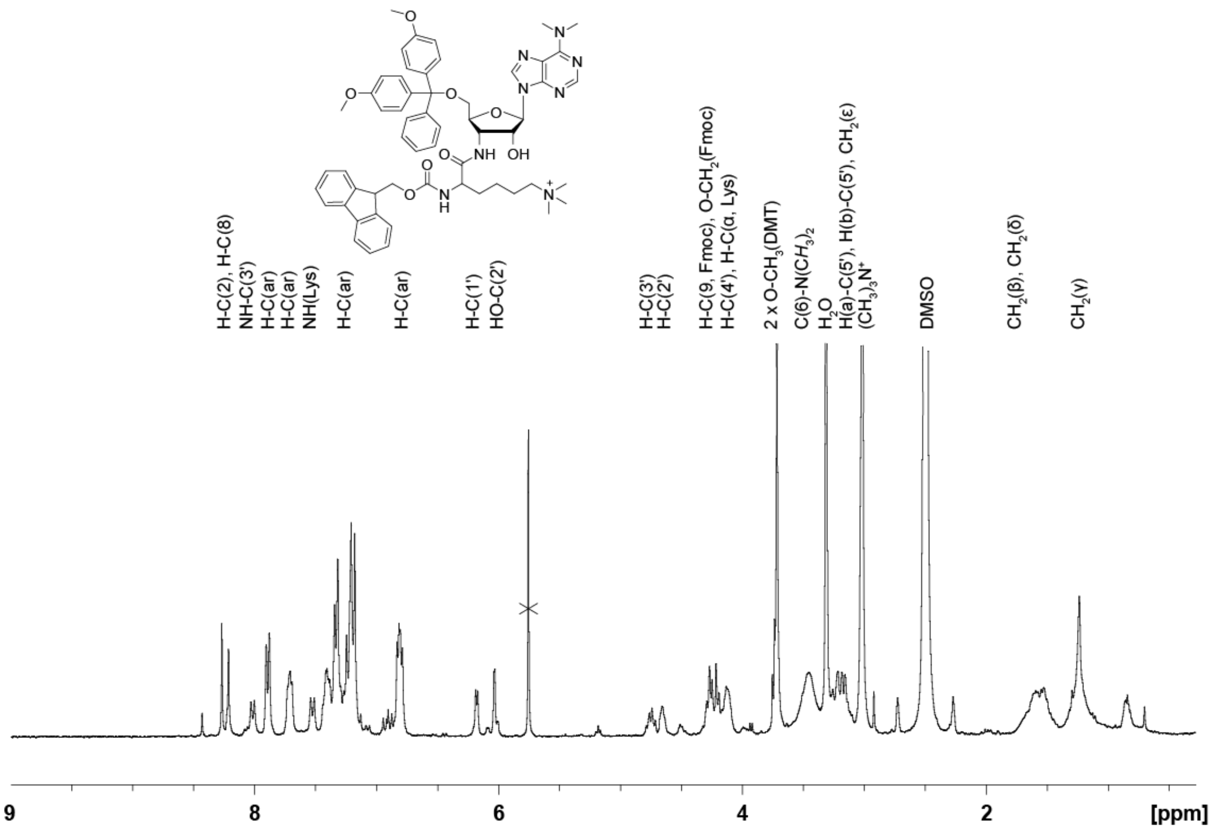

Supplement: Supplementary data — 1H and 13C NMR spectra for compounds 2 to 9. [file mmc1.pdf]
